# Treatment patterns and clinical outcomes in patients with metastatic triple-negative breast cancer: a large-scale data analysis using the Japanese claims database

**DOI:** 10.1007/s10549-024-07273-2

**Published:** 2024-05-05

**Authors:** Takayuki Kimura, Tomoko Takami, Yi Piao, Ioanna Ntalla, Shigehira Saji

**Affiliations:** 1 Medical Affairs, Gilead Sciences K.K., Tokyo, Japan; 2 Real-World Evidence, Gilead Sciences Europe Ltd., Stockley Park, Uxbridge, UK; 3 Department of Medical Oncology, Fukushima Medical University, Fukushima, Japan

**Keywords:** Metastatic triple-negative breast cancer, Treatment pattern, Claims database, Medical cost, Line of therapy, Real-world data

## Abstract

**Purpose:**

This study evaluated treatment patterns and clinical outcomes among patients with metastatic triple-negative breast cancer (mTNBC) in real-world clinical settings in Japan.

**Methods:**

The treatment patterns, time to next treatment or death (TTNTD), time to treatment discontinuation, adverse events of interest, and medical costs of treating patients with mTNBC in first-, second-, and third-line settings were investigated using data of patients meeting the inclusion criteria between January 2017 and March 2022 in a Japanese medical claims database. The treatment regimens for mTNBC were defined according to the Japanese Breast Cancer Society Clinical Practice Guidelines.

**Results:**

In this study, 2236 patients with mTNBC (median age 66.0 years; 99.8% female) were included in the first-line cohort. Of these, 46.6% and 20.8% were included in the second- and third-line cohorts, respectively. The two most frequently used treatments were capecitabine (19.1%) and S-1 (tegafur–gimeracil–oteracil) (14.5%) in the first-line cohort, eribulin (18.3%) and bevacizumab/paclitaxel (14.4%) in the second-line cohort, and eribulin (19.4%) and bevacizumab/paclitaxel (17.5%) in the third-line cohort. The TTNTD shortened as the line of therapy progressed (median 8.0, 6.5, and 5.2 months for the first-, second-, and third-line treatments, respectively). Nausea/vomiting and neutropenia/leukopenia occurred in 62.8% and 18.3% of all patients, respectively. The medical total costs per day were 6.7, 10.2, and 12.9 thousand yen during the first-/second-/third-line treatments, respectively.

**Conclusion:**

This study provides insight into current treatment patterns for mTNBC in Japan. The cost–benefit balance worsens with later-line treatment and a high unmet need for mTNBC drug treatment remains.

**Supplementary Information:**

The online version contains supplementary material available at 10.1007/s10549-024-07273-2.

## Introduction

Triple-negative breast cancer (TNBC), defined by the lack of tumor cell expression of the estrogen receptor (ER), progesterone receptor (PR), and human epidermal growth factor receptor type 2, HER2 [1], accounts for approximately 15% of invasive breast cancers [2–4] and is associated with aggressive tumor biology and poor prognosis. An observational cohort study in Japan reported 13.4 and 12.7 months as the median overall survival (OS) in patients with locally advanced or metastatic TNBC (mTNBC) receiving first- and second-line therapy, respectively [5]. The result of the study suggests that there is a high unmet medical need for Japanese patients with mTNBC.

Chemotherapy has been the main treatment for mTNBC. As first-line treatment options for mTNBC, some agents have recently been approved such as programmed death-1 (PD-1) inhibitors in combination with chemotherapy and poly(adenosine diphosphate-ribose) polymerase inhibitors (PARPis) monotherapy in Japan. According to the NCCN Clinical Practice Guidelines in Oncology (NCCN Guidelines®) [6], combination therapy with pembrolizumab is recommended as the first-line treatment in patients with PD-L1-positive mTNBC regardless of germline breast cancer susceptibility gene (*BRCA*) mutation status. Similarly, PARPis and platinum-based chemotherapy are the preferred regimens in patients with PD-L1-negative and germline *BRCA1/2-*mutated mTNBC, while systemic chemotherapy is recommended in patients with PD-L1-negative and germline *BRCA1/2*-wildtype mTNBC. As for the second-line treatment, PARPis are the preferred regimens in patients with germline *BRCA1/2*-mutated mTNBC. Systemic chemotherapy or sacituzumab govitecan is recommended for treatment regardless of any subtype of mTNBC. Sacituzumab govitecan is approved for second-line and later treatment of mTNBC in multiple countries [7]. Fam-trastuzumab deruxtecan-nxki is recommended as second-line therapy in patients with germline *BRCA1/2*-wildtype and hormone receptor (HR)-negative and HER2 immunohistochemistry (IHC) 1+ or 2+/in situ hybridization (ISH) negative mBC. As third-line therapy and beyond, systemic chemotherapy and targeted agents based on specific genetic mutations are recommended. In the European Society for Medical Oncology (ESMO) guidelines [8], chemotherapy in combination with an immune checkpoint inhibitor (ICI) is recommended as first-line therapy in patients with PD-L1-positive mTNBC. Approximately 40% of patients with TNBC possess PD-L1-positive tumors [9]. Similarly, olaparib and talazoparib are recommended in patients with PD-L1-negative and *BRCA1/2*-mutated mTNBC, while taxane monotherapy is recommended as the preferred option in patients with PD-L1-negative and *BRCA 1/2*-wildtype mTNBC. The guidelines have also recommended sacituzumab as the preferred treatment option for second-line treatment; after progression, all chemotherapy recommendations for HER2− disease also apply for TNBC, such as eribulin, capecitabine, and vinorelbine. In the Japanese Breast Cancer Society Clinical Practice Guideline [10], ICI plus chemotherapy for patients with PD-L1-positive mTNBC have been recommended as first-line therapy. If anthracyclines or taxanes have not been used in perioperative chemotherapy, the standard of care is to administer anthracyclines or taxanes as first-line or second-line chemotherapy for HER2-negative metastatic breast cancer (mBC), respectively. The Japanese guidelines also recommend PARPis as treatment options for patients with *BRCA1/2*-mutated, HER2-negative mTNBC who have previously been treated with chemotherapy, and single-agent chemotherapy for patients with PD-L1-negative, *BRCA1/2-*wildtype mBC. Although ICIs and PARPis have expanded the treatment options for patients of certain subtypes or those having certain biomarkers, chemotherapy remains the standard of care for most patients with mTNBC, and there is a need for therapeutic advances in mTNBC.

Although limited treatment options and poor prognoses for mTNBC have been reported [11], evidence regarding the real-world effectiveness of each line of therapy is limited, particularly in Japan.

In a Japanese observational study in the clinical setting, combination chemotherapy in the first line of treatment showed a higher overall response rate (62.2% for first line vs. 45.1% for second line) and longer progression-free survival (PFS, 9.3 months for first line vs 7.2 months for second line) compared to those showed in the second line of treatment in patients with mTNBC, thereby indicating the importance of optimal treatment choice in each line for patients with mTNBC [5].

Given mTNBC’s aggressive nature, poor prognosis, and limited treatment options, it is important to understand the real-world effectiveness and safety of treatment regimens and patient characteristics.

Therefore, this study evaluated the treatment patterns, time to next treatment or death (TTNTD), time to treatment discontinuation (TTD), adverse events (AEs) of interest, and medical costs among patients with mTNBC in real-world healthcare settings using the Japanese medical claims database.

## Materials and methods

### Overview

This was an observational, retrospective, cohort study using the Japanese medical claims database provided by Medical Data Vision Co., Ltd. (MDV; Tokyo, Japan). Data in this large-scale medical claims database are anonymized and contains unique Japanese diagnosis procedure combination (DPC)/per-diem payment system data, similar to diagnosis-related groups/prospective payment system (DRG/PPS) data. As of September 2021, MDV has accumulated data from more than 38 million patients and 23% of the acute care hospitals in Japan [12].

### Study design and population

Patients with mBC were identified based on the following inclusion criteria: (1) having received a new prescription for any breast cancer treatment regimen, except for hormonal therapy, that were defined as drugs indicated for breast cancer in Japan (excluding adjuvant and neoadjuvant chemotherapy) between January 2017 and March 2022 and (2) diagnosis with breast cancer in the same month as the first-line index date or the month before. Among patients with mBC, the study population with mTNBC were identified by excluding those who did not have HER2, ER, or PR tests before the first-line index date, had a prescription of hormonal therapy or anti-HER2 drugs during the data period, or did not have a 3-month look-back period before the first-line index date.

The first-line regimen was defined as the first regimen administered after mTNBC diagnosis. The index date of the first-line regimen was defined as the start date of the first-line regimen and the first-line cohort was defined as the patient population followed up from the first-line index date; similar definitions were applied to the second- and third-line regimens, index dates, and cohorts. Treatment regimens were classified as either anthracycline-containing, taxane-containing [including taxane-based regimens containing ICI, e.g., atezolizumab/nab-paclitaxel, pembrolizumab/(nab-)paclitaxel], or other regimens. The ICIs were included in the taxane-containing category as the number of patients treated with these regimens was too small to be defined as a separate category. The details of the classification are defined in Online Resource 1.

The exposure period was defined from the first-/second-/third-line index date to the exposure end date. The exposure end date was defined as the earliest date out of the study end date (30 September 2022, the last date of available data), date of death, date of the last prescription of first-line regimen (or second/third-line regimen) considering the grace period, date of drug prescription for breast cancer not included in the first-line regimen (or second/third-line regimen), and date of the last hospital visit.

The grace period was 3 months, regardless of the regimen. The gap period was defined as the interval between the prescription dates for each regimen. If the gap period exceeded the grace period, the date of the grace period that elapsed from the previous prescription date was defined as the date of the last prescription. The follow-up period was defined as the period from the index date of each line until the end of exposure, including the censored date or hospital death. A minimum follow-up period of 180 days was allowed in this study.

### Patient characteristics

Patient characteristics in the first-line cohort were summarized by the category of regimens: all, anthracycline-containing, taxane-containing, and other regimens. The information on age, sex, Charlson comorbidity index, medical history, and malignancy other than breast cancer was obtained from the database. The terms “Lymph node metastasis,” “Respiratory and digestive organ metastasis,” and “Other and unspecified site metastasis” were defined based on the ICD-10 diagnosis codes “Secondary and unspecified malignant neoplasm of lymph nodes,” “Secondary malignant neoplasm of respiratory and digestive organs,” and “Secondary malignant neoplasm of other and unspecified sites,” respectively.

### Outcomes

TTNTD was defined as the time (in months) from the first-/second-/third-line index date until the next treatment or death, whichever occurred first. Patients who were still alive at the study end date or whose exposure ended when the gap period exceeded the grace period were censored at the exposure end date.

TTD was defined as the time (in months) from the first-/second-/third-line treatment start date until discontinuation due to any cause, start of next line, or death. Patients who were still on their first-/second-/third-line treatment, were alive at the study end date, or were lost to follow-up were censored at the last confirmed activity date.

### AEs of interest

The AEs of interest assessed in this study included nausea/vomiting, neutropenia/leukopenia, febrile neutropenia, anemia, diarrhea, interstitial lung disease (ILD), and peripheral neuropathy. The frequencies of these AEs were identified by the disease diagnosis codes (ICD-10 codes) in combination with the treatment prescribed for the AE. The ICD-10 codes and the treatments used are listed in the Online Resource 2. A diagnosis was considered an AE of interest if it occurred during the follow-up period in the same month as the start of the corresponding treatment.

### Medical cost

Medical remuneration points are recorded in a medical claims database. Each cost during the first-/second-/third-line treatment and the cumulative costs from the first-line to the end of third-line treatment were calculated for each patient. Costs per day were also calculated and divided by the follow-up period. The three medical cost subtypes included cost limited to any prescribed drugs (drug cost), cost limited to inpatient claims (hospitalization cost), and cost limited to outpatient claims (outpatient cost).

### Statistical analysis

Statistical analyses were performed using SAS, version 9.4 (SAS Institute, Inc., Cary, NC, USA). Continuous variables were reported as median and interquartile range. Categorical variables were summarized as the number and proportion of the total study population and subgroups. Missing data were not imputed.

For treatment patterns, number of patients and percentages per first-/second-/third-line regimen combination category were calculated. The results were illustrated in a Sankey diagram.

For TTNTD and TTD, the median time to event and 95% confidence intervals (CIs) plus 25% and 75% quantiles were estimated. The results were depicted graphically using Kaplan–Meier curves, plotting the survival rate against time.

Frequency distributions were provided for AEs of interest (nausea/vomiting, neutropenia/leukopenia, febrile neutropenia, anemia, diarrhea, ILD, and peripheral neuropathy) in overall and by age (< 65 or ≥ 65 years).

For medical costs (total costs, total costs per day, drug cost, drug cost per day, hospitalization cost, hospitalization cost per day, outpatient cost, and outpatient cost per day) during the first-/second-/third-line treatments, descriptive statistics (median and interquartile range) were calculated.

Subgroup analysis was performed on treatment pattern by age (< 65 or ≥ 65 years) and by medical history of adjuvant or neoadjuvant chemotherapy at the first-line index date.

## Results

### Patient selection

**Fig. 1:**
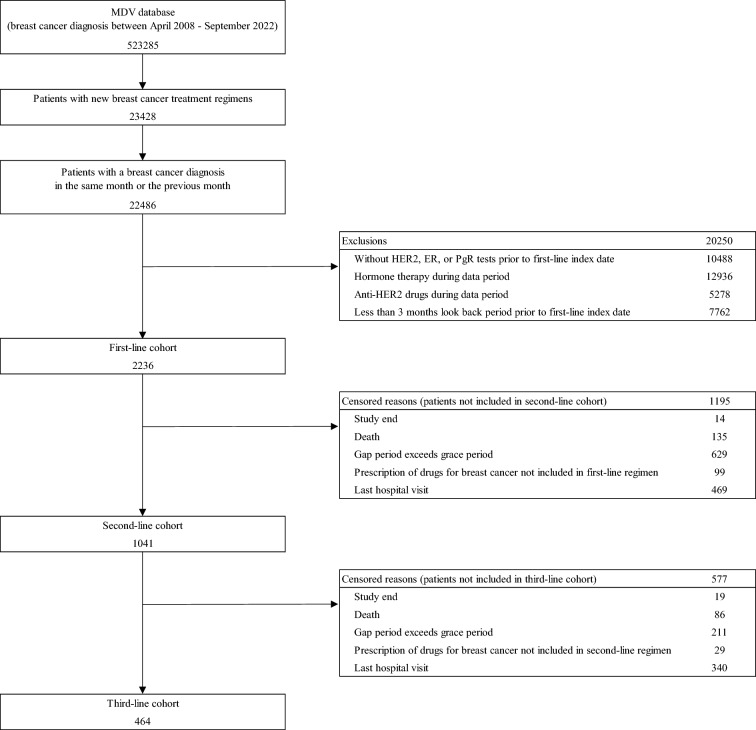
Patient selection. The data of patients diagnosed with breast cancer between April 2008 and September 2022 were extracted from the MDV database. Patients were eligible if they had received a new prescription for any breast cancer treatment regimen (excluding adjuvant and neoadjuvant chemotherapy) between January 2017 and March 2022. If the first regimen prescription date was within 3 months after surgery, the regimen was defined as adjuvant chemotherapy. If the date of the last prescription of the regimen was within 3 months before surgery, the regimen was defined as neoadjuvant chemotherapy. If an anthracycline (doxorubicin/cyclophosphamide, or epirubicin/cyclophosphamide, or epirubicin/fluorouracil/cyclophosphamide) and a taxane (paclitaxel, docetaxel, or nab-paclitaxel) were prescribed sequentially at an interval of ≤ 6 weeks, these regimens were defined as a series of neoadjuvant or adjuvant therapies. *MDV* Medical Data Vision, HER2, human epidermal growth factor receptor type 2, *ER* estrogen receptor, *PR* progesterone receptor

The data of 523,285 patients diagnosed with breast cancer between April 2008 and September 2022 were extracted from the MDV database. Of these, 23,428 patients received their first-line treatment regimens for breast cancer and 22,486 patients had a breast cancer diagnosis in the same or previous month. A total of 2236 met the eligibility criteria and were included in the first-line cohort of mTNBC. Among these, 1041 (46.6%) and 464 (20.8%) were subsequently treated with a second- and third-line treatment and were included in the second-line and third-line cohorts, respectively (Fig. 1).

### Patient characteristics

Patient characteristics in the first-line cohort are shown in Table 1. Of all 2236 patients included in the first-line cohort, 436 (19.5%), 672 (30.1%), and 1128 (50.4%) patients received anthracycline-containing, taxane-containing, and other regimens, respectively. For ICIs, 59 (2.6%) patients received atezolizumab/nab-paclitaxel and 0 (0.0%) patients received pembrolizumab/(nab-)paclitaxel. The median age was 66.0 years in all patients and > 60 years in each regimen group.

**Table 1:** Patient characteristics (first-line cohort)

Parameters	All		Anthracycline-containing regimens		Taxane-containing regimens		Other regimens	
	*N*	%	*N*	%	*N*	%	*N*	%
Total	2236		436		672		1128	
Age (years)								
Median	66.0		61.0		64.0		68.0	
Q1–Q3	53.0–73.0		50.0–70.0		52.0–72.0		55.5–76.0	
Age category (years)								
< 65	1053	47.09	248	56.88	346	51.49	459	40.69
≥ 65	1183	52.91	188	43.12	326	48.51	669	59.31
Sex								
Male	4	0.18	1	0.23	1	0.15	2	0.18
Female	2232	99.82	435	99.77	671	99.85	1126	99.82
Charlson comorbidity index (points)								
Median	8.0		3.0		8.0		8.0	
Q1–Q3	2.0–9.0		2.0–8.0		3.0–9.0		3.0–9.0	
Medical history								
Surgery	1567	70.08	182	41.74	448	66.67	937	83.07
Adjuvant chemotherapy	689	30.81	39	8.94	255	37.95	395	35.02
Anthracycline-containing regimens	366	16.37	9	2.06	172	25.60	185	16.40
Taxane-containing regimens	368	16.46	29	6.65	108	16.07	231	20.48
Other	195	8.72	6	1.38	56	8.33	133	11.79
Neoadjuvant chemotherapy	558	24.96	13	2.98	161	23.96	384	34.04
Anthracycline-containing regimens	508	22.72	9	2.06	153	22.77	346	30.67
Taxane-containing regimens	467	20.89	11	2.52	108	16.07	348	30.85
Radiation therapy	715	31.98	59	13.53	190	28.27	466	41.31
Malignancy other than breast cancer	1420	63.51	209	47.94	421	62.65	790	70.04
Malignant neoplasm of stomach	107	4.79	4	0.92	41	6.10	62	5.50
Malignant neoplasm of bronchus and lung	97	4.34	8	1.83	30	4.46	59	5.23
Lymph node metastasis	915	40.92	137	31.42	253	37.65	525	46.54
Respiratory and digestive organ metastasis	569	25.45	60	13.76	228	33.93	281	24.91
Other and unspecified site metastasis	527	23.57	57	13.07	202	30.06	268	23.76

The median Charlson comorbidity index was 8.0 for all patients and 3.0, 8.0, and 8.0 for patients who received anthracycline-containing, taxane-containing, and other regimens, respectively.

The proportion of patients with a medical history of adjuvant and neoadjuvant chemotherapies was 30.8% and 25.0% for all patients, 8.9% and 3.0% for patients with anthracycline-containing regimens, 38.0% and 24.0% for patients with taxane-containing regimens, and 35.0% and 34.0% for patients with other regimens, respectively. A smaller percentage of patients aged ≥ 65 years (15.8%) received neoadjuvant therapy than patients < 65 years (35.2%), while the percentage of adjuvant therapy was similar between age groups (Online Resources 3, 4).

### Treatment patterns

**Fig. 2:**
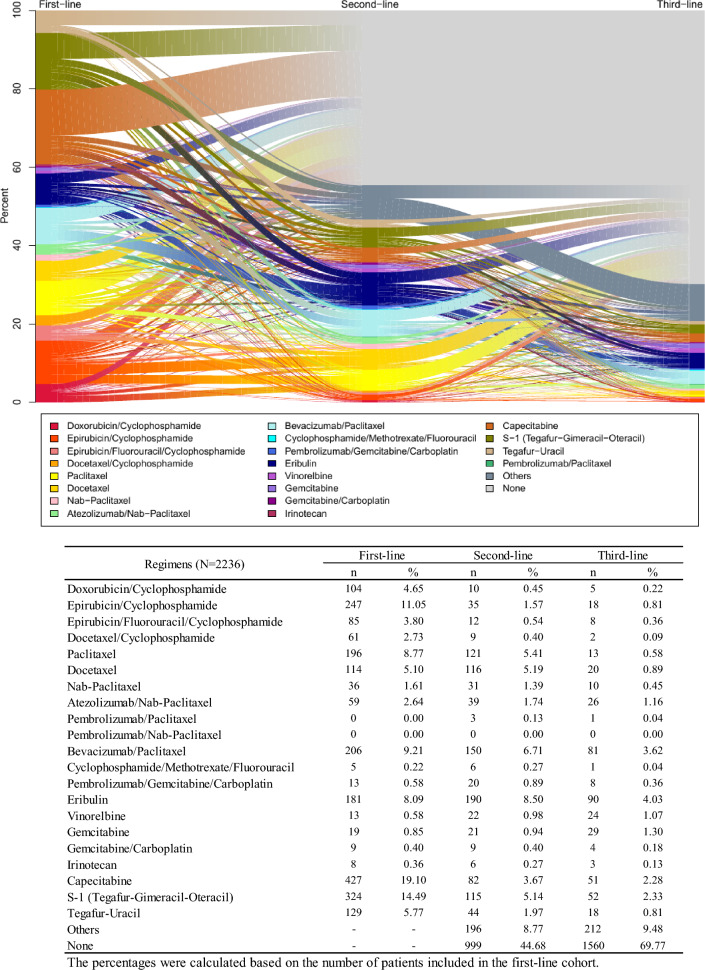
Treatment patterns across the lines of therapies (first-/second-/third-line cohorts). The changes in regimen from the first-line to the third-line therapies are illustrated in a Sankey diagram. Patients who have no record of treatment regimen for mTNBC (Online Resource 1), but received other regimen in second-line or third-line cohort were shown as “others” and those who have no record of treatment for mTNBC were shown as “none.” S-1 is a combination of three pharmacological compounds, namely tegafur, gimeracil, and oteracil

For the 2236 patients who were eligible for this study, the most frequently used treatment regimen was capecitabine (19.1%) as the first-line treatment, eribulin (18.3%) as the second-line treatment, and eribulin (19.4%) as the third-line treatment (Fig. 2; Table 2).

**Table 2:** Most commonly used regimens in each line of therapy

First line (*N* = 2236)			Second line (*N* = 1041)			Third line (*N* = 464)		
Regimes	*n*	%^a^	Regimes	*n*	%^a^	Regimes	*n*	%^a^
Capecitabine	427	19.10	Eribulin	190	18.25	Eribulin	90	19.40
S-1 (Tegafur–Gimeracil–Oteracil)	324	14.49	Bevacizumab/Paclitaxel	150	14.41	Bevacizumab/Paclitaxel	81	17.46
Epirubicin/Cyclophosphamide	247	11.05	Paclitaxel	121	11.62	S-1 (Tegafur–Gimeracil–Oteracil)	52	11.21
Bevacizumab/Paclitaxel	206	9.21	Docetaxel	116	11.14	Capecitabine	51	10.99
Paclitaxel	196	8.77	S-1 (Tegafur–Gimeracil–Oteracil)	115	11.05	Gemcitabine	29	6.25
Others	836	37.39	Others	349	33.53	Others	161	34.70

^a^The percentages were calculated based on the number of patients included in each cohort as denominator

The most common treatment sequence from first- to second-line and from second- to third-line therapy was bevacizumab/paclitaxel to eribulin and eribulin to bevacizumab/paclitaxel, respectively. The most common treatment sequence from first- through third-line therapy was the sequence of S-1 (tegafur–gimeracil–oteracil) to bevacizumab/paclitaxel to eribulin (Online Resource 5).

In patients with adjuvant or neoadjuvant, the most common regimens were capecitabine in the first-line treatment and eribulin in the second-line treatment (Online Resource 6), while in patients without adjuvant nor neoadjuvant, the most common regimens were epirubicin/cyclophosphamide and docetaxel, respectively (Online Resource 7).

Among the patients in the first-line cohort, S-1 (20.3%) and tegafur–uracil (9.4%) were more frequently used in patients aged ≥ 65 years, but epirubicin/cyclophosphamide (8.4%) and bevacizumab/paclitaxel (6.3%) were less frequently used compared to that in patients aged < 65 years (Online Resources 8, 9).

### TTNTD/TTD

**Fig. 3:**
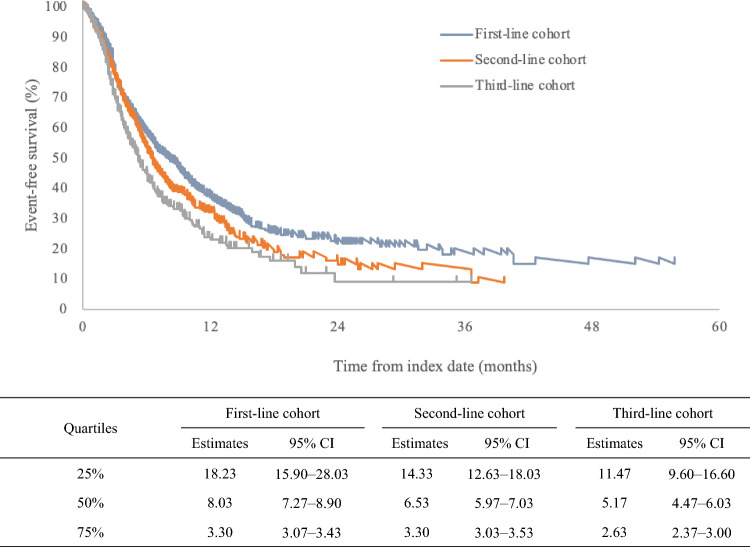
Time to next treatment or death. Time to next treatment or death (TTNTD) was defined as the time (in months) from the first-/second-/third-line index date until the next treatment or death, whichever occurred first. TTNTD included the index date and event date; TTNTD = (event date − index date) + 1. Similarly, if no event was observed during the follow-up period, TTNTD = (end of the follow-up period for the patient − index date) + 1. The results were depicted graphically using Kaplan–Meier curves, plotting the event-free survival rate against time. Kaplan–Meier estimates and the two-sided 95% CIs are shown. TTNTD shortens and diminishes in effectiveness as the therapy progresses. *CI* confidence interval

The median (95% CI) TTNTD was 8.0 (7.3–8.9) months in the first-line cohort, 6.5 (6.0–7.0) months in the second-line cohort, and 5.2 (4.5–6.0) months in the third-line cohort (Fig. 3).

The median (95% CI) TTD was 3.3 (3.1–3.6) months, 3.3 (3.1–3.7) months, and 3.8 (3.0–4.3) months, respectively (Online Resource 10).

### AEs of interest

Nausea/vomiting and neutropenia/leukopenia occurred in 62.8% and 18.3% of all patients, respectively. The proportions of nausea/vomiting (94.1%, 92.3%, and 83.3%), neutropenia/leukopenia (44.5%, 47.0%, and 47.6%), and febrile neutropenia (11.8%, 14.0%, and 14.3%) were high in patients treated with anthracycline-containing regimens (doxorubicin/cyclophosphamide, epirubicin/cyclophosphamide, and epirubicin/fluorouracil/cyclophosphamide, respectively). In contrast, the proportion of these events were low in patients treated with “other regimens” (i.e., capecitabine, S-1, and Tegafur–Uracil, Table 3). The proportions of neutropenia/leukopenia in patients treated with the docetaxel/cyclophosphamide regimen (59.7%) and the proportion of peripheral neuropathy in patients treated with the paclitaxel regimen (16.4%) were the highest among all regimens, respectively.

**Table 3:** AEs of interest (overall; pooled AEs in the first-line, second-line, and third-line cohorts)

	*N*	Nausea/vomiting		Neutropenia/leukopenia		Febrile neutropenia		Anemia		Diarrhea		ILD		Peripheral neuropathy	
		*n*	%	*n*	%	*n*	%	*n*	%	*n*	%	*n*	%	*n*	%
Total	3741	2348	62.8	683	18.3	158	4.2	353	9.4	311	8.3	62	1.7	366	9.8
Doxorubicin/Cyclophosphamide	119	112	94.1	53	44.5	14	11.8	19	16.0	8	6.7	4	3.4	7	5.9
Epirubicin/Cyclophosphamide	300	277	92.3	141	47.0	42	14.0	36	12.0	10	3.3	2	0.7	11	3.7
Epirubicin/Fluorouracil/Cyclophosphamide	105	88	83.8	50	47.6	15	14.3	15	14.3	4	3.8	0	0.0	5	4.8
Docetaxel/Cyclophosphamide	72	60	83.3	43	59.7	10	13.9	4	5.6	3	4.2	1	1.4	8	11.1
Paclitaxel	330	254	77.0	65	19.7	6	1.8	41	12.4	20	6.1	12	3.6	54	16.4
Docetaxel	250	207	82.8	109	43.6	17	6.8	21	8.4	16	6.4	6	2.4	22	8.8
Nab-Paclitaxel	77	48	62.3	12	15.6	3	3.9	14	18.2	7	9.1	0	0.0	11	14.3
Atezolizumab/Nab-Paclitaxel	124	84	67.7	10	8.1	4	3.2	11	8.9	7	5.6	7	5.6	10	8.1
Pembrolizumab/Paclitaxel	4	4	100.0	0	0.0	0	0.0	1	25.0	0	0.0	1	25.0	0	0.0
Pembrolizumab/Nab-Paclitaxel	0	–	–	–	–	–	–	–	–	–	–	–	–	–	–
Bevacizumab/Paclitaxel	437	348	79.6	48	11.0	13	3.0	43	9.8	25	5.7	8	1.8	60	13.7
Cyclophosphamide/Methotrexate/Fluorouracil	12	10	83.3	4	33.3	1	8.3	1	8.3	2	16.7	0	0.0	0	0.0
Pembrolizumab/Gemcitabine/Carboplatin	41	38	92.7	11	26.8	4	9.8	12	29.3	2	4.9	0	0.0	5	12.2
Eribulin	461	337	73.1	83	18.0	15	3.3	36	7.8	27	5.9	9	2.0	56	12.1
Vinorelbine	59	36	61.0	6	10.2	2	3.4	7	11.9	3	5.1	0	0.0	4	6.8
Gemcitabine	69	51	73.9	3	4.3	1	1.4	8	11.6	2	2.9	1	1.4	6	8.7
Gemcitabine/Carboplatin	22	18	81.8	5	22.7	2	9.1	5	22.7	1	4.5	0	0.0	1	4.5
Irinotecan	17	15	88.2	2	11.8	1	5.9	4	23.5	6	35.3	0	0.0	1	5.9
Capecitabine	560	153	27.3	13	2.3	3	0.5	20	3.6	85	15.2	3	0.5	45	8.0
S-1 (Tegafur–Gimeracil–Oteracil)	491	190	38.7	21	4.3	4	0.8	47	9.6	77	15.7	4	0.8	42	8.6
Tegafur–Uracil	191	18	9.4	4	2.1	1	0.5	8	4.2	6	3.1	4	2.1	18	9.4

*N* number of patients pooled in the first-/second-/third-line cohorts, *n* number of patients with AEs pooled in the first-/second-/third-line cohorts, *%* percentage of *n* to *N*, *AE* adverse event, *ILD* interstitial lung disease

Incidence of nausea/vomiting was lower in patients aged ≥ 65 than in those < 65 years. The incidence of neutropenia/leukopenia and febrile neutropenia in patients ≥ 65 was slightly lower than in those < 65 years (Online Resources 11, 12).

### Medical cost

The median total costs per day was 6.7, 10.2, and 12.9 thousand yen during the first-/second-/third-line treatments, respectively. The median drug costs per day were 2.8, 4.8, and 6.1 thousand yen during the first-/second-/third-line treatments, respectively. The median outpatient costs per day were 3.3, 5.1, and 5.7 thousand yen during the first-/second-/third-line treatments, respectively (Table 4).

**Table 4:** Medical cost

Items	First-/second-/third-line cohort	First-line cohort	Second-line cohort	Third-line cohort
N	2236	2236	1041	464
Total cost				
Median	1444.3	782.3	980.3	1096.7
Q1–Q3	561.7–3483.0	409.5–1795.3	430.4–2055.1	426.3–2325.6
Total cost per day				
Median	7.5	6.7	10.2	12.9
Q1–Q3	2.8–15.5	2.5–15.4	3.8–19.9	4.6–21.4
Drug cost				
Median	619.6	388.1	470.1	499.7
Q1–Q3	245.9–1,937.6	184.3–778.1	193.6–1,259.3	175.0–1,414.5
Drug cost per day				
Median	3.3	2.8	4.8	6.1
Q1–Q3	1.3–9.1	1.2–8.9	1.6–13.0	1.8–13.1
Hospitalization cost				
Median	227.0	0	0	0
Q1–Q3	0.0–1,057.2	0.0–557.8	0.0–567.3	0.0–681.3
Hospitalization cost per day				
Median	0.9	0	0	0
Q1–Q3	0.0–5.1	0.0–4.4	0.0–4.9	0.0–7.0
Outpatient cost				
Median	782.9	505.0	585.0	591.2
Q1–Q3	328.9–2101.0	240.4–985.1	250.1–1393.4	207.0–1575.4
Outpatient cost per day				
Median	3.7	3.3	5.1	5.7
Q1–Q3	1.7–8.8	1.6–8.3	2.1–12.9	2.4–14.0

Unit: 1,000 yen. Each cost during the first-/second-/third-line treatment and the cumulative costs from the first-line to the third-line treatment were calculated for each patient. Costs per day were also calculated and divided by the follow-up period

## Discussion

This study provides the first evidence from a multicenter assessment of real-world treatment patterns and outcomes among patients with mTNBC in Japan. Few reports have investigated the treatment patterns for mTNBC in clinical practice in Japan. In Taiwan, the majority of mTNBC patients receive combination therapy as first-line treatment [13]. In contrast, approximately 65% of the patients with mTNBC received monotherapy as the first-line therapy in this study, indicating the proportion of patients who receive monotherapy as first-line therapy for mTNBC in Japan is similar to that in western countries [14, 15].

In this study, 2236 patients with mTNBC were included in the first-line cohort. At the level of individual regimens, capecitabine and S-1 monotherapy were the two most common regimens in first-line treatment, while 20% of patients were treated with anthracycline-containing regimens and 30% were treated with taxane-containing regimens.

Regarding treatment patterns by the presence or absence of perioperative therapy, < 5% of patients with history of perioperative therapy were treated with anthracycline in the first-line treatment; in contrast, approximately one-third of the patients with no history of perioperative therapy were using anthracyclines. According to the Japanese guideline [10], anthracycline regimens are recommended as first- or second-line chemotherapy if they have not been used in perioperative chemotherapy. Hence, the study results suggest that patients were treated with anthracycline regimen in perioperative chemotherapy and may not be eligible for anthracycline regimen in metastatic setting.

In this study, the two most common regimens in the first-line therapy were capecitabine and S-1. These regimens are weakly recommended as first-line treatment in breast cancer clinical practice guidelines. An overseas study showed that cisplatin combined with nab-paclitaxel or paclitaxel is the preferred first-line treatment for mTNBC [16]. This difference between Japan and other countries in the commonly used regimen is most likely due to the difference in the treatments covered by insurance. In this study, eribulin was also the most used drug in the second- and third-line cohorts. Eribulin was shown to prolong OS more than did capecitabine in the second- and later-line setting and is recommended in the guideline for patients treated with anthracyclines and taxanes [17]. Regarding age-specific treatment patterns, S-1 was used more frequently in patients aged ≥ 65 years as the first-line treatment than in those aged < 65 years. S-1 is an oral fluoropyrimidine agent consisting of tegafur (a prodrug of fluorouracil), gimeracil, and oteracil, which is approved for treatment of various cancer types and widely used in Japan [18]. The study showed S-1 has a low incidence of myelosuppression-induced side effects, such as neutropenia. Considering the less frequent AEs of S-1, safety profile might be one of the reasons for treatment choice for older patient.

Regarding the TTNTD, the length decreased with advancement in the line of treatment. Given that TTNTD is often used as a surrogate endpoint for PFS in real-world data, these results are similar to the prospective observational study of bevacizumab combined with paclitaxel in patients with locally advanced or metastatic TNBC, where the median PFS was 9.3 months in the first-line cohort and 7.2 months in the second-line cohort, respectively [5]. However, there was no difference between the lines of treatment regarding the TTD. This suggests that, although there is no change in treatment tolerability in the different lines, the treatment effect seems to weaken as the line progresses.

For AEs of interest, the proportions of nausea/vomiting, neutropenia/leukopenia, and febrile neutropenia in “other regimens” were much lower than those in the anthracycline-containing regimens. This was considered to be one of the reasons that capecitabine and S-1 were the commonly used regimens as the first-line therapy, especially in older patients.

Regarding medical expenses, there was a tendency for costs to increase toward the later lines. As the therapy progresses, the usage rate of relatively inexpensive regimens (doxorubicin/cyclophosphamide, epirubicin/cyclophosphamide, capecitabine, S-1) decreased, while the usage rate of relatively expensive regimens (bevacizumab/paclitaxel, atezolizumab/nab-paclitaxel, eribulin) increased (usage rates were calculated using the patient population included in each cohort as the denominator) contributing to the increase in medical costs as the treatment course progresses. Although public health insurance covers most medical expenses in Japan, long-term treatment costs affect patients’ lives. Thus, treatment costs are a major concern for patients with breast cancer, especially in later line of treatment. The cost effectiveness of each sequence should be further investigated to support the treatment strategy.

Based on the observation collected, the treatment for mTNBC was selected generally according to the treatment guideline in clinical practice in Japan. However, some patients were treated with chemotherapy regimen with low recommendation level. In addition, late-line treatments tend to be less effective and increase the burden of medical expenses. It suggests a high unmet medical need for new treatment options; thus, better risk–benefit profile is necessary.

This study was based on health insurance claims and DPC data. Therefore, some limitations should be considered. Due to the absence of biomarker data for ER, PR, and HER2, patients with TNBC were identified by the absence of hormonal therapy and anti-HER2 drugs. Patients with other breast cancer subtypes may have been included in the study population. HER2 expression has been reported to change during treatment; however, this was not considered in our study because it was not captured in the database. In addition, there may be off-label use of some drugs for breast cancer in Japan. Moreover, owing to unmeasured confounding factors, the possibility of residual confounders cannot be ruled out. Another limitation was that the MDV database has no data of performance status or stage. Therefore, we could not consider important confounders and effect modifiers in this study. The MDV database also does not cover hospital-to-hospital linkages because it is a hospital-based database. Thus, events outside the hospital were not captured. In addition, considering the observation that the median TTNTD is eight months in the first-line cohort, a minimum follow-up period of 180 days might not be optimal for some patients to capture the treatment patterns of the second-line or later therapies. AEs that occurred after discontinuation of a regimen were not collected as they were not in the scope of this study. Medical costs associated with each treatment sequence have not been analyzed in this study and remains a topic for future exploration. Lastly, the findings of this study may deviate from actual practice because the prescription of the regimen is considered an exposure. However, this is unlikely to be a problem because most patients are treated in hospitals.

## Conclusion

This study provided insight into the current patterns of drug treatment for mTNBC in Japan. This suggests that the TTNTD shortens as the therapy progresses. However, the later the line of therapy, the more the cost tends to increase, thus suggesting that the cost–benefit balance worsens with later-line treatment, and there is still a high unmet need for mTNBC treatment.

### Supplementary Information

Below is the link to the electronic supplementary material.

Supplementary file1 (XLSX 2101 kb)

## Data Availability

The data used in this study were provided by the MDV Co., Ltd. under license to Gilead Sciences, Inc. Similar to the consent to participate, because this research used anonymized processed information, the consent was not required.

## Abbreviations

mTNBC: Metastatic triple-negative breast cancer
TTNTD: Time to next treatment or death
TNBC: Triple-negative breast cancer
ER: Estrogen receptor
PR: Progesterone receptor
HER2: Human epidermal growth factor receptor type 2
OS: Overall survival
PD-1: Programmed death-1
PARPis: Poly(adenosine diphosphate-ribose) polymerase inhibitors
NCCN: National Comprehensive Cancer Network
PD-L1: Programmed death-ligand 1
BRCA: Breast cancer susceptibility gene
ESMO: European Society for Medical Oncology
ICI: Immune checkpoint inhibitor
mBC: Metastatic breast cancer
PFS: Progression-free survival
TTD: Time to treatment discontinuation
AEs: Adverse events
DPC: Diagnosis procedure combination
ILD: Interstitial lung disease
MDV: Medical Data Vision
CIs: Confidence intervals
